# Amalgam

**Published:** 1884-10

**Authors:** S. C. G. Watkins


### AMALGAM.

BY S. C. G. WATKINS, D. D. S.

READ BEFORE THE NEW JERSEY STATE DENTAL SOCIETY AT ITS FOURTEENTH ANNUAL MEETING, AT ASBURY PARK, JULY, 1884.

There is an article used by the Dental Profession to a great extent which has received much condemnation, and yet hundreds of the practical men in our profession who have patience and skill—the time, the courage and the honor to do good and careful work with a material less dazzling than gold—look upon Amalgam as a God-send to the dentist and the patient. Some have condemned it to such a degree that they have put themselves on record as the lamented Dr. Webb did, when he said before the First District Society in New York that "he had not used Amalgam for six years, and would not use it again as long as he lived."

This reminds me of a doggerel verse that I found in a dental journal some years since :—

> " But when it came to fitting the stump
> With a proxy limb, then flatly and plump
> She spoke in the spirit olden ;
> She couldn't, she shouldn't, she wouldn't have wood,
> Nor a leg of cork, if she never stood,
> And she swore an oath, or something as good,
> The proxy limb should be golden." *

So Amalgam is discarded entirely, and by the golden enthusiast it is insisted that if a tooth is worth filling at all it is worth filling with gold, and perhaps they put upon their cards " nothing but gold used," thereby ignoring the other materials, especially Amalgam, which, by careful manipulation, may prove so useful and beneficial.

Many of the advocates of heavy gold disapprove the use of Amalgam. They put heavy gold in all kinds of cavities and teeth, malleting with almost force enough to break down a prison door, and building up the contour over frail walls. What condition must the weak structure be in after such treatment ? And again, one-third of the good tooth substance is cut away in order to get at the cav-

* The quotation is from Thomas Hood's " Miss Kilmanugg."

ity so that gold can be malleted home, as it is claimed that all gold should be malleted, even the first piece; they must either place the gold in the cavity without seeing just where it is put, and not be sure that the cavity is well filled around the cervical wall, or else cut away an unnecessary amount of good tooth substance. Undoubtedly Amalgam is preferable in either of these cases.

Much of this radical gold furor, I firmly believe, arises from a pecuniary motive. The idea is prevalent that gold is more valuable than the other metals, therefore fillings made of gold are more valuable than those made of other materials, and gold fillings are inserted without regard to the anatomical or physiological condition of the teeth; many cases exist where a large gold filling would be positively injurious, causing the death of the pulp, discoloration of the tooth, abscess, loss of tooth, or necrosis.

The insertion of large gold fillings in molars is so wearing upon both patient and operator, and so expensive, that the patient is completely disheartened, and refrains from visiting a dentist again for several years, the result being that there is positive loss, both to patient and operator. Amalgam would be much better in such cases. Our practice to be successful must be based upon a sound philosophy; that is, upon the knowledge of those principles and laws that govern the materials or agents with which we operate, and also those upon whom we operate. We fill teeth that we may arrest decay, prevent pain, assist in mastication, preserve the anatomical contour of the face, lend help to the vocal organs that they may produce certain sounds, as well as to earn a livelihood for our families and a reputation for ourselves. If the circumstances under which we fill teeth are favorable they will greatly assist us in bringing out good results, and the opposite will do much to retard, unless there shall be brought to bear such helps as will completely neutralize all opposition. The first thing to consider in the filling of teeth is the condition of the case in hand, through a faithful diagnosis. To diagnose a case for filling we should notice the condition of the structure, whether healthy or unhealthy, its location and its importance to its possessor. When its unhealthy condition is changed to a healthy one and all decayed matter removed, cavity shaped, walls formed, edges trimmed and polished properly, we are ready for the filling. Now the question arises, "What shall I fill with?" After a careful and conscientious examination of the surrounding circumstances I should a few years ago have said gold, in most cases, like a good many of my friends in the profession. I received my dental education at the Boston College, and many prejudices against Amalgam were inculcated into my training, and they were generally entertained at that time by most of the able men of the profession, very few having the courage to acknowledge that they used it, or to defend its good qualities. I have seen several cases where Amalgam has stood the wear and tear of forty years. And again, I have seen beautiful gold fillings from the hands of our most skillful operators fail under favorable circumstances.

With regard to the different kinds of Amalgam, there is little use in speaking. I prefer the alloys of Hood of Boston, Lawrence of Boston, and Dawson of New York. I have used these Amalgams with great success in all conditions of cases in molars. In some cases I have built up the whole crown, where there was but a small rim of the enamel and the roots were hollowed out like funnels. Many would have condemned them for extraction; others, passionate builders of gold crowns, would have persuaded the patient to endure the prolonged operation and pay for it in proportion. Such teeth, when properly prepared and filled, with the Amalgam thoroughly impacted, are good for many years of service without periostitis setting in as soon as the work is completed, which in many cases would be the result after such a cavity had been filled with gold. The worst thing that can be said against Amalgam is its color, and that in two of the varieties which I have named is not very objectionable for the molars.

Unfortunately, we are not all born wealthy. Some cannot pay for gold fillings. Others are not willing to pay for them under all circumstances; and again, some have not the physical strength to endure the insertion of a large gold filling in the posterior surface of a molar, or in many cases in the bicuspids, and sometimes a very small oral cavity will prevent. There is a class of cavities which I particularly wish to call attention to; viz., posterior approximal cavities in the molars, or where a posterior and an anterior approximal come in close contact. In such cases most Amalgam fillings are failures, not because of any fault in the Amalgam or any fault in the tooth structure, but on account of gross carelessness on the part of the operator. In all cases of approximal fillings, whether of gold, amalgam, oxy-phosphate, or gutta percha, the rubber dam should be placed upon the teeth, the cavity properly prepared with under-cuts from all sides where there is sufficient tooth substance to warrant it, and the periphery thoroughly trimmed and rounded, so that there will be no frail edges, especially cervical-approximal. Then the cavity should be washed out with either an antiseptic or an embalmer. Prof. Mayr's theory is that bi-chloride of mercury, or corrosive sublimate is the best antiseptic we have for such cases, as in very small quantities it will thoroughly destroy the bacteria, while pure carbolic acid, as we have known for a long time, will embalm the bacteria by coagulating the albumen in the canaliculi, thereby sealing the entire surface and preventing the lower organisms from forming under the filling, thus causing decay. For the same purpose, some prefer the thin gutta percha varnish, or copal-ether varnish, which answers a very good purpose if care is taken not to allow it to remain around the periphery of the cavity. In large quantities it is sometimes well to more than half or two-thirds fill with oxy-phosphate or oxychloride of zinc, allowing the filling to extend cone-shaped in the centre, leaving space for the Amalgam to be placed upon the top of it.

The Amalgam should be mixed very dry and placed in the cavity in small particles, and thoroughly packed around the walls with the same care that is necessary in gold filling. If the Amalgam is so dry that it is difficult to work it, the instruments should be heated so that the material will pack more thoroughly, and draw most of the mercury to the surface, when it can be wiped out. When the tooth is thoroughly filled, with suitable instruments made very thin and curved to suit the different cases, trim away all excess of material till you come to the perfectly filled edges of the cavity, then draw back and forth over the filling bibulous paper folded three or four times, or fine linen tape. By so doing you will make smooth surfaces, and fill any imperfection which there may be, leaving the filling exactly flush with tooth substance. Some claim that they can fill such cases properly without the dam, and finish up the surface perfectly with silk floss drawn back and forth over the filling and under the edge of the gum, thus removing all excess of material. This I claim to be impossible if the Amalgam has been properly condensed. Many cases that have fallen under my eye are found to be filled too full, the amalgam projecting beyond the edges of the cavity, in some cases the filling extending beyond the cervical wall an eighth of an inch, sending out ragged edges from all sides excepting the grinding surface. I have a number of such fillings in my possession which were inserted by some of the good men in our profession, and some by those not so promising. After turning out such work is it any wonder that Amalgam is thus denounced, and all praise and glory given to gold? When I see such fillings I cannot help but feel that the dentist charged but a dollar, and then was afraid that he was giving his patient a dollar's worth. In preparing cavities for amalgam fillings in the grinding surfaces, a mistake is often made by not cutting out the small fissure decay all the way to the end, in which case the decay extends, and it is not long before the tooth requires refilling. Sometimes in filling such cavities the Amalgam is allowed to project beyond the cavity, and overlap the enamel, and in a very short time it chips off and leaves ragged edges to catch the food.

Again, I find Amalgam an excellent material to partially fill a cavity in the posterior surface of the bicuspids or molars, and then cover with gold. I place a very thin band of copper around the tooth and ligate it well with floss; it being very soft, the floss will draw it close around the neck of the tooth. If the tooth is so shaped that the copper will not give to it, you can burnish it down to the cervical edge so as to get a perfect contour. Mix the Amalgam very dry, and pack so as to condense thoroughly, until the cavity is about three-quarters full; then cut away the filling until it is about one-half full, and finish with gold. The first few pieces will be embedded in the Amalgam, and you can build from that and form your contour perfectly. When the copper matrix is removed you will find very little trimming to be done, and in my opinion it will be a better filling in that position than if it were all gold. In filling children's teeth, the temporary as well as the six-year molars, I find Amalgam invaluable. When a child is nervous and impatient it is difficult to keep it quiet long enough to use any other material; again, the density of these teeth is not as great as in the teeth of older persons, therefore gold should not be used in such cases, and this much abused amalgam is better than any other preparation for such cavities in the crowns and approximal surfaces of the molars.

The rubber dam is an invaluable agent in the insertion of Amalgam fillings, although many claim that its use is not necessary; but I have noticed in my own fillings, as well as those of others, that the most durable and best were those where the dam was used. My patients often remark to me that they have never had the dam used for such fillings, and it is a rare thing to see a perfect approximal filling made of Amalgam for this very reason.

