# A Provoking Blunder

**Published:** 1884-10

**Authors:** 


A PROVOKING BLUNDER.

When the first form of this number was presented to us for final revision, we wrote, as plain as the nose upon the compositor's face, a correction in the foot-note upon page 552. "Miss Kilman*segg* and her Wooden Leg" is the name of the poem referred to, but when the form was all printed we found it as it appears—Kilman*ugg*. Ugh! as if we did not know a name with which we have been familiar from childhood.

